# Fundamental Frequency Layer-Wise Optimization of Tow-Steered Composites Considering Gaps and Overlaps

**DOI:** 10.1007/s42496-024-00212-w

**Published:** 2024-04-22

**Authors:** A. Pagani, A. Racionero Sánchez-Majano, D. Zamani, M. Petrolo, E. Carrera

**Affiliations:** MUL2 Lab, Department of Mechanical and Aerospace Engineering, Politecnico di Torino, Turin, Italy

**Keywords:** Variable stiffness composites, Variable-angle tow, Unified formulation, Defect layer method, Defect modeling, Surrogate optimization

## Abstract

The advent of Automated Fiber Placement (AFP) in aerospace composites lay-up and manufacturing has allowed orientations to vary along pre-defined curved directions rather than being forced to remain constant within the lamina. These composites are called Variable Angle Tow (VAT) or Variable Stiffness Composites (VSC). Despite the enhancements in mechanical performance offered by VAT, constraints from the manufacturing process hinder their full potential. This paper explores the effect of primary defects, i.e., gaps and overlaps, on optimal design and fundamental frequency optimization. For doing so, the Carrera Unified Formulation (CUF) and the Defect Layer Method (DLM) are integrated directly into the optimization process to provide an efficient and cost-effective framework for modeling the structural behavior and manufacturing process of VSCs. Particular attention is given to manufacturing and tow-steering simulation to quantify and map defects for each laminate layer. This research serves a dual purpose: (i) examining the impact of process-induced defects on achieving an optimal design and (ii) exploring how the choice of structural theory may affect the optimal solution.

## Introduction

The latest advances in composite manufacturing techniques, such as automated fiber placement (AFP) [1, 2], and automated tape laying (ATL) [3] have enabled the design of curvilinear fiber paths instead of the traditional straight fiber lay-up, which exhibits constant mechanical properties within each layer. Known as variable stiffness composites (VSC) or variable angle tow (VAT), these laminates enhance the design possibilities and create new design options not conceivable with traditional composites [4].

In the last decades, due to their promising capabilities, it has been imperative to mechanically characterize VAT laminates in terms of buckling and vibration response [5]. For instance, Lee and Harper [6] evaluated the improved buckling performance of tow-steered laminates with a cutout. Gürdal et al. [7] found that axial stiffness is decoupled from buckling behavior by investigating the change in in-plane stiffness during buckling as the fiber orientation was varied. Moreover, Vescovini et al. [8] introduced a numerical approach using the Ritz method in combination with first-order theories to model the pre-buckling and buckling of VSCs, which was shown to accurately calculate the buckling condition at a lower computational cost than most finite element based methods. On the other hand, Akhavan and Ribeiro [9] analyzed the natural frequencies and modal forms of VSCs using both first-order shear deformation theory (FSDT) and third-order shear deformation theory (TSDT). It has been demonstrated that the significant advantage of using curved fibers is the increased flexibility that can be efficiently utilized to adjust frequency and mode shapes. Pereira et al. [10] carried out a modal characterization study of VAT laminates, focusing on damping, using a model that combined the semi-analytical Rayleigh–Ritz approach, classical laminated plate theory (CLPT), and the strain energy method. They were able to estimate the exact damping capacity of each mode. Another important aspect was explored in the study of Stodieck et al. [11] where the use of tow-steered composites for the tailoring of the aeroelastic behavior of a rectangular wing was modeled utilizing the Rayleigh–Ritz method and strip theory aerodynamics. Also, a method for predicting the aeroelastic flutter state for flat and curved VAT plates under supersonic flow was proposed in the study of Sharma et al. [12]. It was concluded that VAT laminates have the potential for improved design compared to traditional unidirectional composite laminates.

In contrast to the CLPT and FSDT models, which are examples of equivalent single-layer (ESL) theories, it is possible to implement layer-wise (LW) models, where each layer is modeled with independent variables (degrees of freedom) and continuity of interlaminar displacements must be imposed at the interface. One of the first attempts to use LW theory to model VAT was made by Demasi et al. [13] in which two-dimensional (2D) ESL, Zig-Zag, and LW theories were presented, and different orders of expansion were implemented using the Carrera Unified Formulation (CUF) to capture the behavior through the thickness accurately. Furthermore, Viglietti et al. [14] proposed a refined one-dimensional (1D) CUF-based model for the free vibration analysis of VSCs. Sánchez-Majano et al. [15] studied the stress distribution of VAT laminates using ESL and LW theories. Furthermore, Pagani et al. [16] conducted nonlinear geometric analyses of VAT plates to examine the vibration in nonlinear equilibrium states. The crucial task of the mesoscale’s mechanical characterization was addressed in Ref. [17, 18]. Specifically, the LW approach was used to describe the layer scale, while a 1D component-wise (CW) method was implemented to characterize the fiber-matrix scale. This approach accurately captured the 3D stress state at different scales. These investigations have concluded that the ESL and LW models are consistent with the results of major commercial software tools for analyzing in-plane stress components in VAT laminates. Nevertheless, refined kinematics were required to predict the out-of-plane stresses accurately.

Defects resulting from the AFP manufacturing process are a critical aspect that characterizes VSCs. Such manufacturing signatures cause discrepancies between the finished component and the original numerical model. As stated in [19], these defects are the main limitation of the AFP technology. The works of Nguyen et al. [20, 21] presented experimental results on the effects of process-induced imperfections when using AFP technology and found a strong influence of gaps on the compressive behavior of composite laminates. At the same time, overlaps improved tensile properties and showed negligible compressive changes. Blom et al. [22] suggested using a denser mesh in areas where the defect is most prevalent to capture the effect of this defect type on mechanical behavior. In contrast, Fayazbakhsh et al. [23] proposed the Defect Layer Method (DLM), which accounts for gaps by scaling the material’s elastic properties and obtains overlaps by adjusting the layer thickness. It was found that despite the number of finite elements involved, the DLM was more accurate in identifying and estimating the area of defects. A method for investigating the fundamental frequency of variable stiffness laminates that combines DLM and CUF has been presented in [24]. Notably, it was observed that the fundamental frequency decreased with gaps but increased with overlaps due to their structural stiffening effect.

As outlined in [25], the future success of utilizing advanced tailoring techniques hinges on developing optimization tools that consider the manufacturing signature. In this regard, Carvalho et al. [26] conducted a study on optimizing the lamination angle to maximize fundamental frequency. They integrated gaps into the optimization process through a modified rule of mixtures, and the optimization problem was solved using a Genetic Algorithm (GA). Nik et al. [27, 28] introduced a multi-objective optimization framework for designing VAT laminates using a surrogate algorithm incorporating an evolutionary model to reduce computational cost. The optimization framework’s main goals were to investigate which parameters influence the optimal solutions set while maximizing the in-plane stiffness and critical buckling load. It was discovered that when the course width remained constant, the defect areas within the laminate were reduced as the number of tows rose. Additionally, adopting a complete gap strategy resulted in a decreased in-plane stiffness and critical buckling load. Conversely, utilizing a complete overlap strategy yielded superior outcomes in both magnitudes. Another important study [29] combined GA with a pattern search, creating a hybrid optimization approach that reduced weight by 31% compared to traditional straight fiber composites. Similarly, Zhao and Kapania [30] used Particle Swarm Optimization (PSO) to optimize the buckling load of a perforated VAT plate, taking into account manufacturing constraints such as maximum curvature and parallel fiber path to reduce the gap and overlap. Catapano et al. [31] suggested a multi-scale two-level optimisation strategy for VAT laminates, considering the manufacturing requirements associated with the Fused Filament Fabrication (FFF) and Continuous Filament Fabrication (CFF) techniques. Notably, the importance of incorporating technological constraints into the design optimisation process was highlighted. Finally, Sánchez-Majano and Pagani [32] implemented a GA where a surrogate model mimicked the objective function to maximize the buckling load and the fundamental frequency of VAT plates. Using CUF to construct the surrogate brought the advantage of obtaining a model whose accuracy was determined by appropriately selecting the order of the structural theory adopted.

The objective of this work is to present an optimization framework that maximizes the first natural frequency of a VAT laminate while considering machine simulation to characterize the manufacturing signature of the AFP process. This is achieved by adjusting the lamination angles $T_0$ and $T_1$ for each layer while considering the impact of defects, such as gaps and overlaps. Additionally, the goal is to evaluate how the selection of a structural theory may impact the optimal solutions identified. Because CUF is used as a generator of structural theories, the selection is made straightforwardly. The paper is structured as follows: Sect. 2 presents the main characteristics of VAT plates and how the defect area is estimated and mapped for each layer; Sect. 3 provides the formal description of CUF and free vibration problem using unified finite elements; Sect. 4 introduces the proposed optimization framework to maximize the fundamental frequency of VSCs; Sect. 5 contains the model verification and optimization results, and Sect. 6 concludes the paper.

## VAT Plates and AFP Machine Simulation

In this work, the fiber orientations in the plane are represented by the notation proposed by Gürdal et al. [7] and reads as follows:

1 $$\begin{aligned} \theta (x') = \phi + T_0 + \frac{(T_1-T_0)}{d}|x'|. \end{aligned}$$

**Fig. 1:**
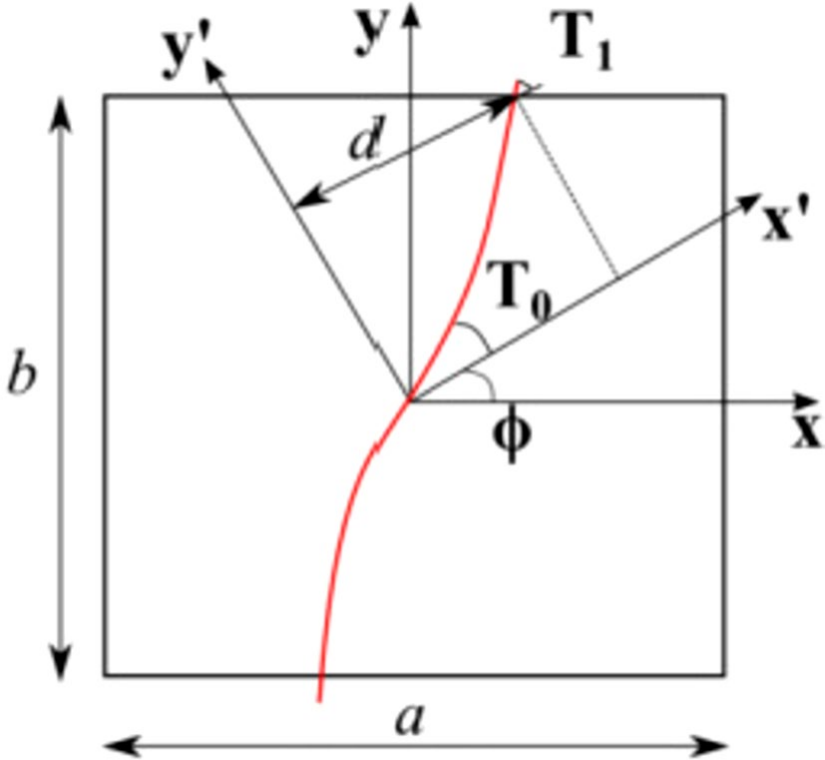
Graphical representation of the fundamental parameters that describe the course of the linearly varying fiber path in the plane

This type of fiber control is known as linear variation, in which the local fiber orientation $\theta$ varies along the direction $x'$. This direction can be at an angle $\phi$ with respect to the *x* axis. The orientation of the fibers at $x' = 0$ is called $T_0$, while the angle at $x' = d$ is called $T_1$. As shown in Fig. 1, the distance *d* represents the length of the fiber between $T_0$ and $T_1$. The coordinate $x'$ can be expressed as $x' = x\cos \phi + y\sin \phi$ according to the global coordinate system $x-y$. Therefore, for the results presented in this study, the $x'$ direction aligns with the global *x* axis, and *d* is equal to *a*/2.

For the representation of the centerline shown in Fig. 1, Eq. (2) is adopted, as it is indicated in Ref. [26].

2 $$\begin{aligned} y(x) = {\left\{ \begin{array}{ll} \frac{a}{2(T_0-T_1)}\{\text {ln}(\cos {T_0})+\text {ln}[\cos {(-T_0+2T_1+\frac{2(T_1-T_0)}{a})}x]\} \hspace{3.5mm} -a\le x \le -\frac{a}{2}\\ \frac{a}{2(T_1-T_0)}\{-\text {ln}(\cos {T_0})+\text {ln}[\cos {(T_0+\frac{2(T_0-T_1)}{a})}x]\} \hspace{12mm} -\frac{a}{2}\le x \le 0\\ \frac{a}{2(T_0-T_1)}\{-\text {ln}(\cos {T_0})+\text {ln}[\cos {(T_0+\frac{2(T_1-T_0)}{a})}x]\} \hspace{16.5mm} 0\le x \le \frac{a}{2}\\ \frac{a}{2(T_1-T_0)}\{\text {ln}(\cos {T_0})+\text {ln}[\cos {(-T_0+2T_1+\frac{2(T_0-T_1)}{a})}x]\} \hspace{6.5mm} \frac{a}{2}\le x \le a\\ \end{array}\right. } \end{aligned}$$

**Fig. 2:**
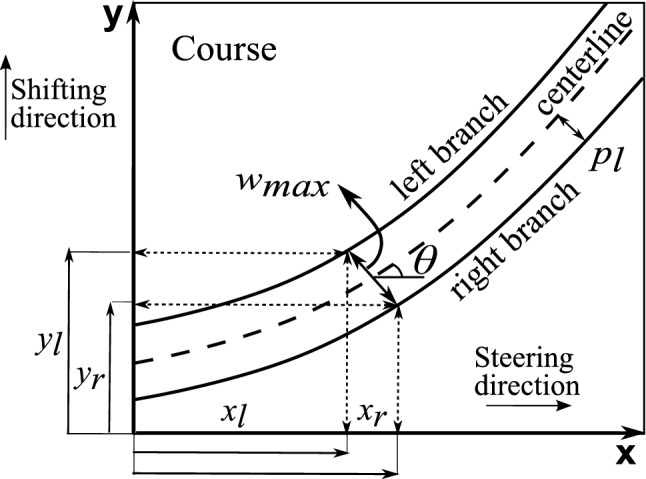
Representation of a single course displaying the AFP parameters used to plot the centerline and the left and right branches

Once the central line is created, it is possible to generate a left and a right branch at an appropriate distance from the central filament by incorporating AFP parameters that emulate the steering process, as seen in Fig. 2. Specifically, $N_t$ represents the number of tows laid down during the steering process. Then, the width of a single tow is defined by $t_w$, which is set to 3.125 mm in this paper. Finally, $r_{min}$ represents the AFP machine’s minimum radius of curvature capability, which is equivalent to 625 mm. After determining the AFP machine parameters, the different courses for each laminate layer are simulated using the geometric parameters shown in Fig. 2. For this purpose, Eq. (3) gives the coordinates in the plane of the left and right branches from the centerline of the fiber path. In detail, the term $x_l$ represents the domain of the left branch of the fundamental fiber, while $x_r$ represents the domain of the right branch. The terms $y_l$ and $y_r$ represent the left and right branches of the fundamental fiber, respectively. The term $p_l$ represents the semi-width of the course and is calculated as $0.5\cdot N_tt_w$.

3 $$\begin{aligned} {\left\{ \begin{array}{ll} x_l = x - p_l\sin \theta (x) \hspace{5em} x_r = x + p_l\sin \theta (x)\\ y_l(x) = y(x) + p_l\cos \theta (x) \hspace{2.5em} y_r(x) = y(x) - p_l\cos \theta (x).\\ \end{array}\right. } \end{aligned}$$

In Ref. [33], different manufacturing constraints are presented to be applied to optimization methods to find solutions feasible by AFP machines. The one used in this study is defined in Eq. (4), which considers the maximum bending radius achievable by the AFP machine.

4 $$\begin{aligned} \kappa = \frac{1}{r} = \frac{2(T_1 - T_0)}{a}\cos \left( (T_1 - T_0)\frac{x}{a/2} + T_0\right) \ \end{aligned}$$

Provided a pair of $T_0$ and $T_1$ for each layer, it is then possible to determine if a design can be effectively manufactured by evaluating the local curvature $\kappa$, which must not exceed the maximum allowable curvature $\kappa _{{\text {lim}}}$.

**Fig. 3:**
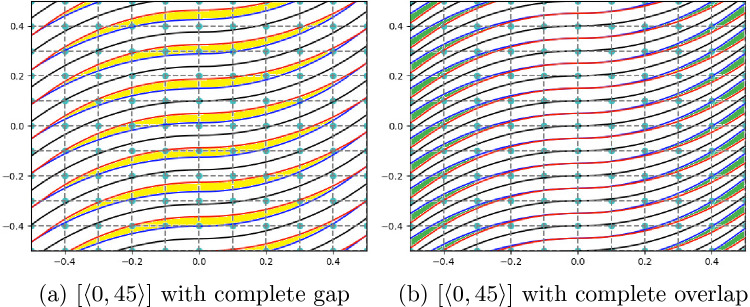
Example of a plate with [$\langle 0,45 \rangle$] stacking sequence with a complete gap (left) and complete overlap (right) implementing the edge tangency and center tangency steering strategy, respectively

**Fig. 4:**
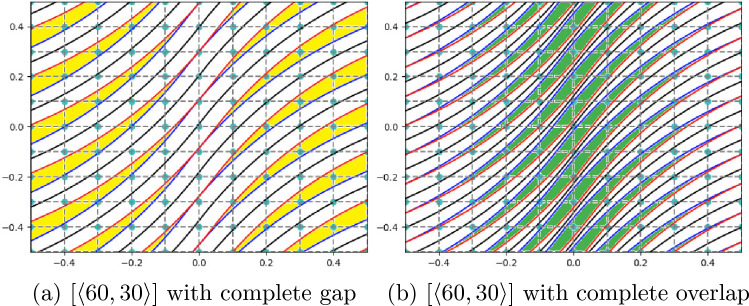
Example of a plate with [$\langle 60,30 \rangle$] stacking sequence with a complete gap (left) and complete overlap (right) implementing the center tangency and edge tangency steering strategy, respectively

The occurrence of gaps and overlaps is influenced by the values of lamination angles $T_0$ and $T_1$, as well as the deposition strategy employed, as shown in Table 1. The laying down of two consecutive courses can be enforced based on a tangency condition at the center of the plate or contact at the edge. In this regard, Fig. 3 illustrates the case of a [$\langle 0,45 \rangle$] plate in which the edge tangency and center tangency strategy between two consecutive courses is employed. When the first strategy is adopted, gaps appear in the center of the plate, as indicated by the yellow areas in Fig. 3a. On the contrary, when using the contact at the center strategy, overlap at the edges is apparent, as highlighted by the green color in Fig. 3b. Figure 4 shows the case of a [$\langle 60,30 \rangle$] plate implementing the previously described strategies. In particular, in this case, where $T_0 > T_1$, the implementation of the contact at the center strategy leads to gaps at the edges of the laminate, as shown by the yellow regions in Fig. 4a. On the other hand, as shown in the green areas in Fig. 4b, the implementation of the edge tangency strategy shows the presence of overlaps in the center of the plate. A summary of the defects that arise depending on the tangency condition and the selection of $T_0$ and $T_1$ is available in Table 1.

**Table 1:** Type and location of defects based on fiber characteristics and manufacturing strategy

Condition	Tangency at the edge	Tangency at the center
$|\cos (T_0)|$ > $|\cos (T_1)|$	Gap at center	Overlap at edges
$|\cos (T_0)|$ < $|\cos (T_1)|$	Overlap at center	Gap at edges

**Fig. 5:**
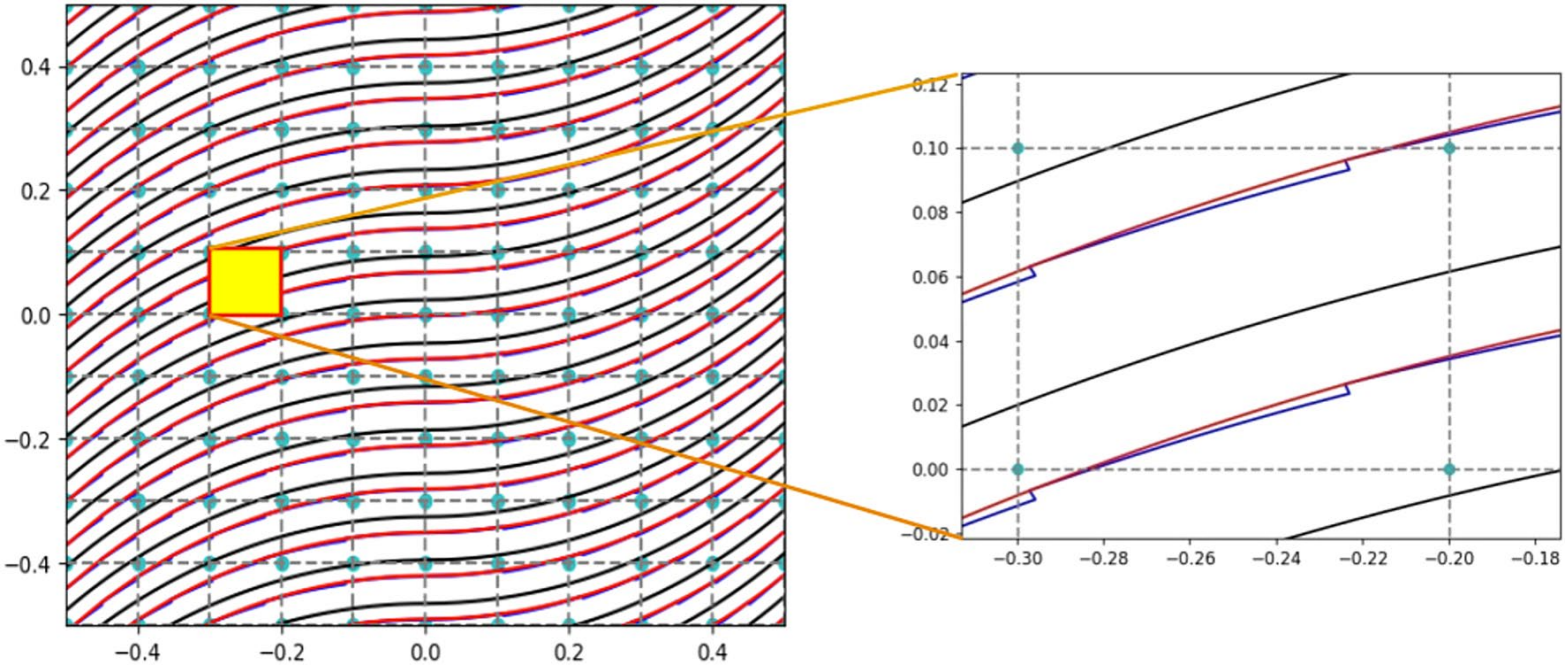
Representation of the gap defect correction strategy over a [$\langle 0,45 \rangle$] ply. The zoomed area shows the triangular gaps generated along the fiber’s direction

Once the centerline has been obtained, the following step fills the entire plate with the appropriate number of linearly varying fiber path repeats to cover it completely. Large imperfections are visible within each layer as the course width remains constant throughout the steering process. The course width must be widened or narrowed when one course intersects another or does not extend to the edge of the previous course to reduce the area of imperfection. Through the application of this technique [34], it has been discovered that imperfections can be reduced by introducing small triangular areas along the entire direction of the fiber path rather than in the center or at the edges of the plate, as can be seen in Fig. 5. The model employed in this work simulates the correction process with a coverage parameter, which establishes the degree of overlap permitted for each course. In particular, with a coverage value of 0%, there will only be gaps, whereas with a value of 100%, there will solely be overlaps.

**Fig. 6:**
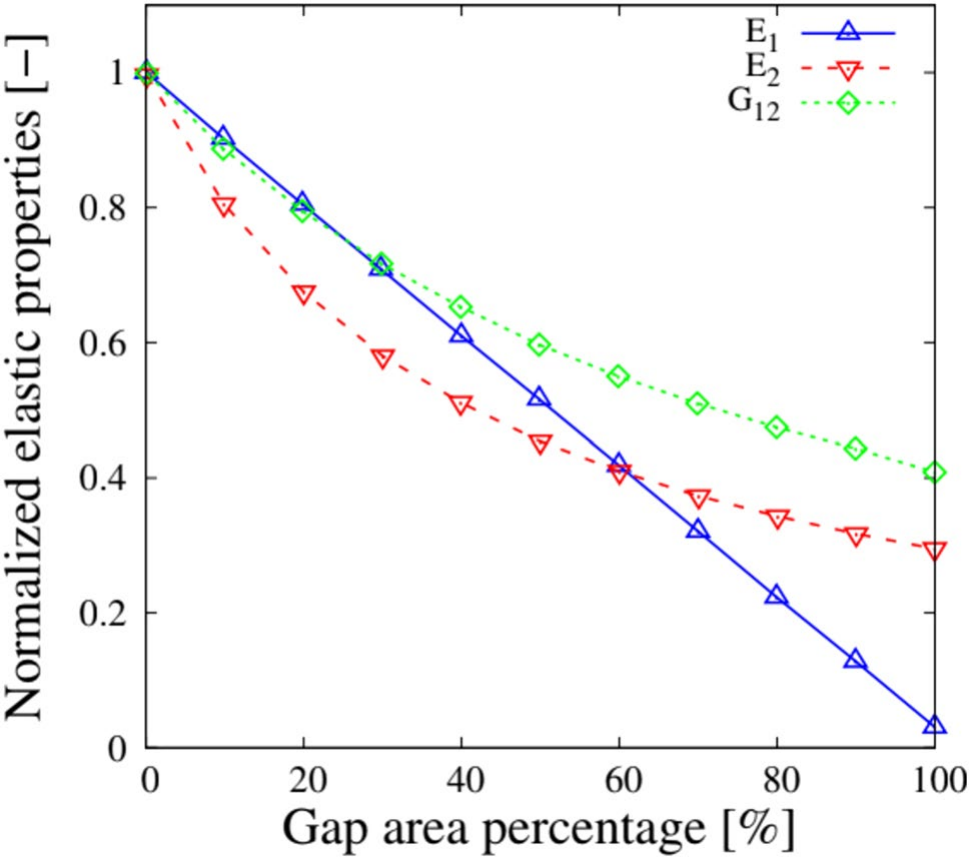
Normalized elastic properties with respect to the gap area percentage. Adapted from [23] with permission from Elsevier

After quantifying and mapping the fabrication flaws, it is necessary to transfer them to the structural model using the DLM mentioned above. This integration is achieved by discretizing the midplane of the plate and modifying the properties of the individual FE based on the calculated percentage of defects. In this way, the only parameter that needs to be adjusted to simulate the presence of flaws resulting from the AFP process is the variation of the material’s elastic properties or thickness. Figure 6 shows the variation in the material’s elastic properties for each element as a function of the gap area, $A_{gap}$. The effective density can also be calculated using the mixing rule as follows:

5 $$\begin{aligned} \rho _{{\text {element}}} = (1 - A_{{\text {gap}}})\rho _{{\text {pre-preg}}} + A_{{\text {gap}}}\rho _{{\text {resin}}} \end{aligned}$$

in which $\rho _{{\text {pre-preg}}}$ and $\rho _{{\text {resin}}}$ are the densities of the pre-impregnated tape and the resin, respectively. In the case of overlaps, the mechanical characteristics of the material are preserved, but the thickness of the FE is locally increased in proportion to the amount of overlap defect, $A_{{\text {overlap}}}$. As stated in [35], due to the compaction pressure in the autoclave, the maximum thickness increase of the ply for this research is limited to 95%, as can be seen in Eq. (6).

6 $$\begin{aligned} t_{{\text {overlap}}} = 0.95 \cdot t_0(1 + A_{{\text {overlap}}}). \end{aligned}$$

## Carrera Unified Formulation CUF

In this paper, a 2D FE within the CUF framework [36] is implemented. According to CUF, the 3D field of displacement is expressed using arbitrary through-the-thickness expansion functions $F_{\tau }(z)$ of the generalized displacements as follows:

7 $$\begin{aligned} \textbf{u}(x,y,z) = F_{\tau }(z)\textbf{u}_{\tau }(x,y) \hspace{2em} \tau = 1,\ldots ,M. \end{aligned}$$

Following Einstein’s notation, the subscript $\tau$ denotes a summation over the *M* expansion terms. The ${\textbf {u}}_{\tau }(x,y)$ represents the generalized displacements in the $x-y$ plane. This paper uses Taylor expansion (TE) to generate an ESL model. Instead, a Lagrange expansion (LE) is employed over the single layers to generate LW models. The continuity of displacements at the layer interfaces is imposed to obtain a Lagrange displacement-based (LD) LW approach, as prescribed in [37]. In this scenario, the expressions TE*n* and LD*n* represent the utilization of Taylor or Lagrange polynomials of *n*-th order, respectively. In addition, *X*LD*n* is used for LW models to indicate the presence of *X* Lagrange polynomials of *n*th order per layer. By coupling the FE method with CUF, the subsequent 3D displacement field is derived:

8 $$\begin{aligned} \textbf{u}(x,y,z) = N_{i}(x,y)F_{\tau }(z){\textbf {q}}_{\tau i} \hspace{2em} i = 1,\ldots ,N_n \hspace{2em} \tau = 1,\ldots ,M \end{aligned}$$

in which ${\textbf {q}}_{\tau i}$ denotes the unknowns vector, and $N_n$ is the number of nodes in each element. This work considered bi-quadratic Q9 FE to be employed as shape functions $N_i(x,y)$.

The governing equations are derived through the principle of virtual displacements (PVD). The problem is described in terms of virtual work using the principle underlying PVD, which, in the case of free vibration analysis, can be expressed as follows:

9 $$\begin{aligned} \delta {\mathcal {L}}_{{\text {int}}} + \delta {\mathcal {L}}_{{\text {ine}}} = 0. \end{aligned}$$

The virtual variations of the internal and inertial forces can be written in the following forms:

10 $$\begin{aligned} \delta {\mathcal {L}}_{{\text {int}}}= & {} \int _V\delta {\varvec{\epsilon }}^{T}{\varvec{\sigma }} dV \end{aligned}$$

11 $$\begin{aligned} \delta {\mathcal {L}}_{{\text {ine}}}= & {} \int _V\rho {\ddot{\textbf{u}}}\delta {\textbf{u}}^TdV. \end{aligned}$$

Equation (10) can be reformulated by employing Eq. (8), the constitutive equation $\varvec{\sigma }=\textbf{C}\varvec{\varepsilon }$, and the geometrical relations between strains and displacements, resulting in

12 $$\begin{aligned} \delta \mathcal {L}_{{\text {int}}} = \delta \textbf{q}_{sj}^T\left[ \int _V\textbf{D}^T(N_jF_s) \mathbf {\widetilde{C}}\textbf{D}(N_iF_{\tau })dV\right] \textbf{q}_{\tau i} = \delta \textbf{q}^T_{sj}\textbf{k}^{ij\tau s}\textbf{q}_{\tau i} \end{aligned}$$

in which $\textbf{k}^{ij\tau s}$ is the 3 $\times$ 3 Fundamental Nucleus (FN) of the stiffness matrix, which is invariant of the order of $N_{i}(x,y)$ shape function and the $F_{\tau }(z)$ theory expansion. The differential operator matrix containing the geometrical relations is expressed by the term $\mathbf {D(\cdot )}$. As described in [38], $\mathbf {\widetilde{C}}$ is the point-wise varying material stiffness matrix defined in the global reference frame, which is calculated as $\mathbf {\widetilde{C}} = \textbf{T}(x,y)\textbf{C}\textbf{T}^T(x,y)$. The $\textbf{C}$ matrix represents the elastic coefficients of the orthotropic material, while $\textbf{T}$ represents the rotational matrix, as explained in Ref. [39]. Emphasis should be placed on the dependency of $\textbf{T}$ on the in-plane coordinates due to the curvilinear fiber paths in VAT. A similar approach is applied to Eq. (11) obtaining:

13 $$\begin{aligned} \delta \mathcal {L}_{{\text {ine}}} = \delta \textbf{q}_{sj}^T\left[ \int _V \rho \textbf{I}F_{\tau }F_{s}N_{i}N_{j}dV\right] \varvec{\ddot{q}}_{\tau i}=\delta \textbf{q}_{sj}^T\textbf{m}^{ij\tau s}\varvec{\ddot{q}}_{\tau i}, \end{aligned}$$

where $\textbf{I}$ stands for the 3 $\times$ 3 identity matrix and $\textbf{m}^{ij\tau s}$ is the 3 $\times$ 3 diagonal FN of the mass matrix, as explained in Ref. [36]. The global element mass and stiffness matrices can be obtained from the FNs based on the given approximation order. The final system is obtained by looping over the number of FE. Thus, the undamped free vibration problem can be expressed as follows:

14 $$\begin{aligned} \textbf{M}\mathbf {\ddot{q}}+\textbf{K}\textbf{q}=0, \end{aligned}$$

where $\textbf{M}$ and $\textbf{K}$ represent the global mass and stiffness matrices, respectively. These matrices are derived by iterating through the FNs using the *i*, *j*, $\tau$, and *s* indices to compute the mass and stiffness matrices for an individual element and assemble them to form the global structure’s matrices. By imposing pure harmonic solution $\textbf{q} = \mathbf {\widetilde{q}}e^{i\omega t}$ Eq. (14) results in the following eigenvalue problem:

15 $$\begin{aligned} \left( \textbf{K} - \omega _{i}^2\textbf{M}\right) \mathbf {\widetilde{q}}_{i} = 0 \end{aligned}$$

in which $\omega _i$ is the *i*-th natural frequency and $\mathbf {\widetilde{q}}_{i}$ represents the *i*th eigenvector.

## Optimization Framework

**Fig. 7:**
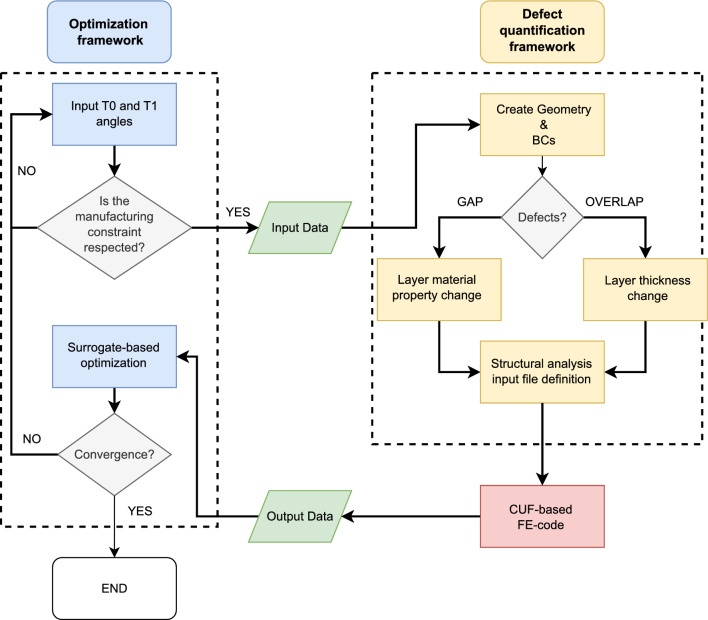
Flowchart of the surrogate-based optimization framework considering the constraint $\kappa _{{\text {lim}}}=1.57 \text {m}^{-1}$ and the quantification of the defects performed by DLM. Finally, the CUF-based FE code is used to calculate the fundamental frequency

Compared to traditional composites, VSCs offer designers a significantly larger design space. Nevertheless, due to the intrinsically inhomogeneous stiffness properties of the computational domain, resulting in higher computational costs (see [40]), it is crucial to develop appropriate and effective optimization and design tools.

In this paper, the objective is to maximize the first fundamental frequency of a benchmark three-layered VAT laminate by varying the lamination angles $T_0$ and $T_1$ for each layer. The optimization problem can then be defined as follows:

16 $$\begin{aligned} \begin{aligned}&\text {maximise} \hspace{10.5mm} f_{1} \\&\text {by varying} \hspace{5mm} -90^\circ \le \textbf{T}_0\le 90^\circ \\&\hspace{24.3mm} -90^\circ \le \textbf{T}_1\le 90^\circ \\&\text {subject to} \hspace{8mm} \kappa \le \kappa _{{\text {lim}}} = 1.57 \hspace{1mm} \text {m}^{-1}, \\ \end{aligned} \end{aligned}$$

where the design variable vectors $\textbf{T}_0$ and $\textbf{T}_1$ are formed by three components: $\textbf{T}_0$=$\{T_0^1$,$T_0^2$,$T_0^3\}$ and $\textbf{T}_1$=$\{T_1^1$,$T_1^2$,$T_1^3\}$. Since the tow width used in this article is equal to 3.125 mm, the maximum curvature of the AFP machine is chosen as the constraint of the optimization problem and is equal to $\kappa _{{\text {lim}}}$ = 1.57 m^-1^, as indicated in [26]. The optimization problem described in Eq. (16) is solved using the framework presented in Fig. 7. Specifically, the MATLAB^©^ environment is used to generate the values of $T_0$ and $T_1$ for each iteration using the *surrogateopt* algorithm [41]. The manufacturing constraint imposed by the $\kappa _{{\text {lim}}}$ parameter is verified first. If the constraint is met, the objective function is then computed. The quantification and mapping of defects for each layer of the laminate have been performed using a Python code, the theoretical basis of which has been explained in Sect. 2. After simulating defects for each layer, input files for the FE analysis are generated by adjusting the material or thickness based on the percentage area of gaps and overlaps, respectively. The objective function is then calculated by solving the problem using unified finite elements, as described in Sect. 3.

The use of a surrogate algorithm, as opposed to a genetic algorithm for instance, offers the advantage of faster execution time when dealing with complex objective functions that require lengthy evaluation at each iteration. To ensure proper functionality of the solver, it is necessary to establish upper and lower bounds for the variables that will be adjusted to determine the function’s minimum. In this case, the lower and upper bounds for $\textbf{T}_0$ and $\textbf{T}_1$ are initially set at -90^∘^ and 90^∘^, respectively, as stated in [26, 30]. The initial sampling procedure for evaluating the objective function is implemented using Latin Hypercube Sampling (LHS). This step is crucial as the objective function is interpolated through *radial basis functions* to create the surrogate. The lowest value obtained by evaluating the objective function at the initial points is referred to as the *incumbent point*. The objective function is then evaluated around this point using the interpolated model to generate *adaptive points*. A fitness parameter, also known as a merit function, is assigned to these points, taking into account the value assumed by the surrogate model evaluation and the distance between the sample points and the points to be evaluated. The minimum fitness parameter determines the point that becomes a new sample point, making it the only adaptive point for which the objective function is evaluated instead of the surrogate model. This effectively adds a new point to the interpolating function. The process is repeated iteratively until convergence, starting with the creation of incumbent points followed by adaptive ones, as described above.

## Numerical Results

### Verification of the CUF-DLM Approach

**Table 2:** Mechanical properties of the prepreg and resin used in [42]

	$E_1$ [GPa]	$E_2$[GPa]	$G_{12}$[GPa]	$G_{13}$ [GPa]	$G_{23}$[GPa]	$\nu _{12}$	$\rho$ [kg/m^3^]
Prepreg	143	9.1	4.82	4.9	4.9	0.3	1500
Resin	3.72	3.72	1.43	1.43	1.43	0.3	1100

The numerical model’s consideration of defects and the manufacturing process simulation are verified against the outcomes reported in [42]. Specifically, symmetrical and balanced sixteen-layer laminate with stacking sequence [$\pm \langle 58, 39 \rangle$]_4s_ was considered. The laminate length-to-thickness ratio *a*/*h* is 200, and the width and length of the plate are *a* = *b* = 1 m. The mechanical properties of the prepreg and resin are listed in Table 2. Both complete gap and complete overlap strategies were implemented with a simply supported boundary condition on all four sides. Table 3 presents the convergence analysis of the FE mesh by varying the number of Q9 quadratic 2D elements while using LD1 kinematics through the thickness for each ply in the case of a complete gap strategy. In this context, the acronym LD1 refers to the LW model using linear Lagrange expansion functions to capture the behavior along the thickness of the plate [37]. The findings suggest that a 6 $\times$ 6 Q9 FE mesh effectively captures the first fundamental frequency. This mesh leads to a relatively low computational effort, facilitating the reduction of the computational burden associated with the optimization process. Nonetheless, a more refined FE mesh would be required if natural frequencies other than the first were considered. The slight difference between the results obtained in Table 3 and Ref. [42] may be due to the different structural theories implemented. In particular, compared to the reference model, which implements a TSDT theory, the results shown in Table 3 involve the use of linear LW structural models in which it is possible to consider the defects layer by layer.

**Table 3:** Mesh convergence analysis using Q9 elements and a complete gap condition

Model	DOFs	$f_1$ [Hz]
Ref. [42]	–	30.95
4 $\times$ 4 Q9-1LD1	4131	32.12
6 $\times$ 6 Q9-1LD1	8619	31.89
8 $\times$ 8 Q9-1LD1	14,739	31.81
10 $\times$ 10 Q9-1LD1	22,491	31.77

LD1 kinematics are implemented through the thickness

**Fig. 8:**
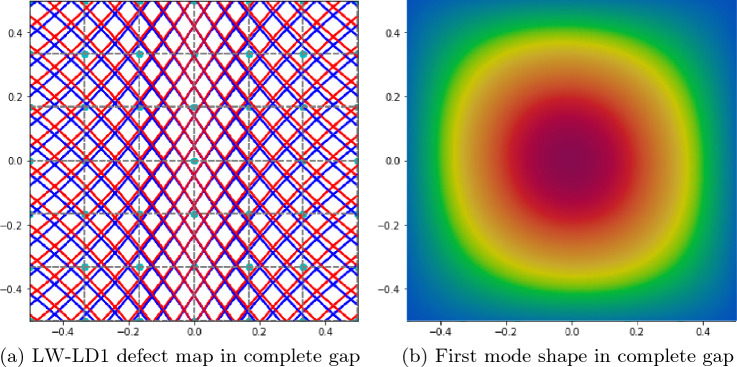
Defect map and first mode shape for complete gap and LD1 kinematics along the thickness

Table 4 presents the impact of the structural theory on the fundamental frequency while implementing a complete gap strategy. A 6 $\times$ 6 Q9 FE mesh was utilized in these models. Varying the expansion functions between TE and LD expansions did not result in any significant fluctuations of the first natural frequency of the laminate. Precisely, it is observed from the table that both the ESL and LW models overestimate the first natural frequency of the laminate. Specifically, it is observed that the highest value is obtained using TE1 structural theory, which is similar to an FSDT model, while employing LW models yields values closer to the reference frequency. This is because the implementation of high-order kinematics along the thickness allows defects to be considered layer-wise. An additional cause for discrepancy between the reference results and those proposed is the different modeling of the fiber path in the plane. Specifically, Ref. [42] considers a plate with a constant curvature fiber path, while a linearly varying fiber path is employed in this work. Figure 8 shows the defect map of the complete gap and LD1 kinematics along the thickness. To achieve the complete gap condition, contact must be imposed at the center of the laminate, as explained in Table 1. This allows defects to be observed at the edges of the laminate. In addition, the first modal form in the complete gap condition for the verification case is shown in Fig. 8.

**Table 4:** Effect of the structural theory on the first fundamental frequencies

Model	DOFs	$f_1$ [Hz]
Ref. [42]	–	30.95
6 $\times$ 6 Q9-TE1	1041	32.17
6 $\times$ 6 Q9-TE2	1521	31.91
6 $\times$ 6 Q9-TE3	2028	31.90
6 $\times$ 6 Q9-1LD1	8619	31.89
6 $\times$ 6 Q9-1LD2	16731	31.89
6 $\times$ 6 Q9-1LD3	24843	31.89

LE and TE expansions are considered through the thickness with a complete gap condition

Table 5 provides the convergence analysis of the FE mesh by varying the number of Q9 elements while using TE3 kinematics through the thickness in the case of a complete overlap strategy. It is observed that as the mesh becomes finer in the plane, the fundamental frequency decreases. This is because a denser mesh can more accurately capture and distribute the defect to the finite elements in the plane. In this instance, it is shown that a 6 $\times$ 6 FE mesh can be acceptable to capture the first natural frequency of the laminate while maintaining a relatively low computational cost.

**Table 5:** Mesh convergence analysis using Q9 elements and a complete overlap condition

Model	DOFs	$f_1$ [Hz]
Ref. [42]	–	36.19
4 $\times$ 4 Q9-TE3	972	37.06
6 $\times$ 6 Q9-TE3	2028	36.64
8 $\times$ 8 Q9-TE3	3468	36.03
10 $\times$ 10 Q9-TE3	5292	35.91

TE3 kinematics are implemented through the thickness

**Fig. 9:**
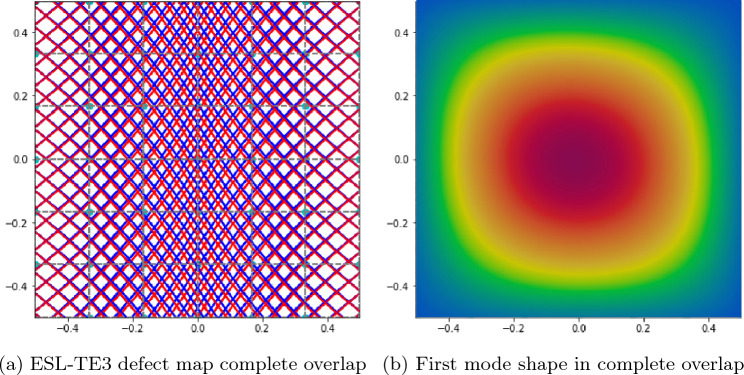
Defect map and first mode shape for complete overlap and TE3 kinematics along the thickness

Table 6 illustrates the effect of the structural theory on the fundamental frequency when a complete overlap strategy is implemented. These models utilized a 6 $\times$ 6Q9 FE mesh. Only structural theories that employ TE expansions through the thickness were selected in the case of complete overlap. Section 2 illustrates that when there are only overlaps, the thickness is subject to a local increase associated with the FE, which is proportional to the amount of defects. This modeling approach for overlaps results in a significant rise in computational expense for CUF-based LW models. This is due to the need to add supporting LD expansions along the *z*-direction to ensure the continuity of the thicknesses of the different AFP machine paths. As a result, overlaps are solely modeled using ESL structural theories. This technique compresses the thickness increments into a single layer, eliminating the need for additional expansions that would overly burden the model’s computational cost. The defect map of complete overlap and TE3 kinematics along the thickness is shown in Fig. 9. To achieve complete overlap, contact must be made at the edge of the laminate, as indicated in Table 1. Defects can be observed at the center of the laminate. Additionally, Fig. 9 displays the first modal shape in the complete overlap condition for the verification case.

**Table 6:** Effect of the structural theory on the first fundamental frequencies

Model	DOFs	$f_1$ [Hz]
Ref. [42]	–	36.19
6 $\times$ 6 Q9-TE1	1014	37.00
6 $\times$ 6 Q9-TE2	1521	36.65
6 $\times$ 6 Q9-TE3	2028	36.64
6 $\times$ 6 Q9-TE4	2535	36.64
6 $\times$ 6 Q9-TE5	3042	36.64

TE expansions are considered through the thickness with a complete overlap condition

### Frequency Optimization Results

Initially, the objective is to illustrate the optimisation of the fundamental frequency of a traditional three-layer straight fiber composite clamped on four edges by varying the lamination angles for each ply. The geometry and mechanical properties of the materials are shown in Table 7. Table 8 shows the optimum angles of the non-steered laminate. The design variables are contained in the vector $\varvec{\theta }=\{\theta ^1,\theta ^2,\theta ^3\}$. The optimization problem is solved using the surrogate-based framework presented in Sect. 4. All structural theories lead to an optimum [$90^\circ ,0^\circ ,90^\circ$] lamination.

**Table 7:** The geometric and material properties of the VAT plate taken into account for the optimization, from [9]

Parameter	Value
$a = b$, m	1
*h*, m	0.01
$E_1$, GPa	173
$E_2 = E_3$, GPa	7.2
$G_{12} = G_{13} = G_{23}$, GPa	3.76
$\nu _{12}$ = $\nu _{13} = \nu _{23}$	0.29
$\rho$, kg/m^3^	1540

**Table 8:** Optimal angles of non-steered laminate by varying structural theory

	ESL-TE1	ESL-TE3	LW-LD1	LW-LD2
$\theta ^1$[^∘^]	90	90	90	90
$\theta ^2$[^∘^]	0.04	$-$0.03	0.04	$-$0.04
$\theta ^3$[^∘^]	90	90	90	90
$f_1$[Hz]	116.77	115.86	116.08	115.86

**Fig. 10:**
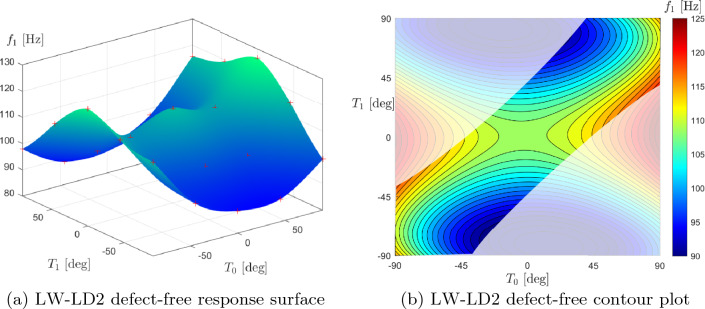
Response surface and contour plot of the fully clamped optimal defect-free plate. The angles $T_0$ and $T_1$ of the second layer were kept fixed at $T_0^2=-50.16^\circ$ and $T_1^2=-36.98^\circ$; while the angles of the first and third layers were changed and assumed $T_0^1$=$T_0^3$ and $T_1^1$=$T_1^3$. The shaded region in the contour plot represents the unfeasible design space

After verifying the defect quantification model and presenting the non-steered results, the aim is to maximize the first natural frequency of a three-layer VAT laminate by adjusting the lamination angles $T_0$ and $T_1$ for each ply. Table 7 shows the plate’s material properties and geometry, taken from Ref. [9]. The optimization outcomes for the defect-free laminate are presented in Table 9, which will serve as a benchmark for cases where defects will be included in the numerical model. In detail, the optimal lamination angles are presented for the different structural theories implemented and where the optimization constraint is $\kappa _{{\text {lim}}}$ is equal to 1.57 m^-1^. Figure 10 displays the contour plot with the limitations due to the maximum bending radius of the AFP machine and the LW-LD2 defect-free response surface. Based on Table 9 findings, it was assumed that $T_0^1$=$T_0^3$ and $T_1^1$=$T_1^3$, and $T_0^2=-50.16^\circ$ and $T_1^2=-36.98^\circ$ were fixed to produce these charts.

**Table 9:** Optimal defect-free design angles varying the structural theory with the optimization constraint $\kappa _{{\text {lim}}}$ = 1.57 m^-1^

	ESL-TE1	ESL-TE3	LW-LD1	LW-LD2
$\langle T_0,T_1 \rangle$ ^1^ [^∘^]	$\langle 88.31,43.35 \rangle$	$\langle 88.25,43.29 \rangle$	$\langle 89.62,44.71 \rangle$	$\langle 88.73,43.79 \rangle$
$\langle T_0,T_1 \rangle$ ^2^ [^∘^]	$\langle -56.84,-15.69 \rangle$	$\langle -34.99,-17.86 \rangle$	$\langle -56.55,-42.80 \rangle$	$\langle -50.16,-36.98 \rangle$
$\langle T_0,T_1 \rangle$ ^3^ [^∘^]	$\langle 87.77,42.95 \rangle$	$\langle 88.38,43.40 \rangle$	$\langle 88.80,43.98 \rangle$	$\langle 88.86,43.92 \rangle$
$f_1$ [Hz]	120.74	119.57	119.35	119.29

**Fig. 11:**
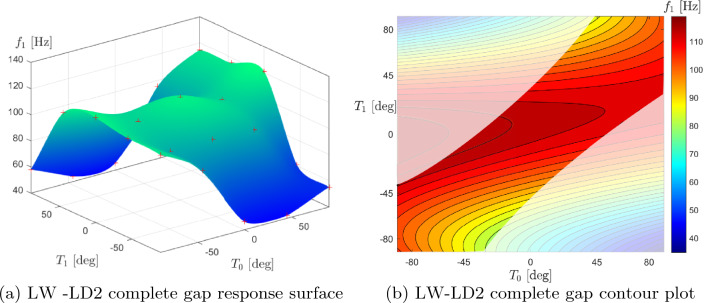
Response surface and contour plot of the fully clamped optimal complete gap plate. The angles $T_0$ and $T_1$ of the second layer were kept fixed at $T_0^2=47.76^\circ$ and $T_1^2=36.46^\circ$; while the angles of the first and third layers were changed and assumed $T_0^1$=$T_0^3$ and $T_1^1$=$T_1^3$. The unfeasible design space is represented by the shaded area in the contour plot

Table 10 shows the optimal angles of $T_0$ and $T_1$ for the three layers of the plate when the complete gap strategy is adopted. Furthermore, to prevent singularities in the model for defect estimation and mapping, it is recommended to restrict the range of the design variables $T_0$ and $T_1$ from -89^∘^ to 89^∘^, as suggested by [26]. In addition, as in the defect-free case, a 6 $\times$ 6Q9 FE mesh was used, which, as mentioned above, was found to be sufficient to capture the first natural frequency of the VAT laminate in the presence of defects. Note that the resin properties from Table 2 are considered in this optimization. Subsequently, a similarity of the optimal angles $T_0$ and $T_1$ as the structural theory varies is also appreciated, as shown in Table 10. Slight variations are observed in the optimum angles for each layer and the maximum achievable natural frequency as the applied structural theory varies. In addition, it is worth noting that for the complete gap strategy, the first and third layers present an almost unsteered design. Figure 11 shows the response surface and the contour plot with the design space restrictions due to the optimisation constraint for the complete gap condition. It is assumed that only the angles of the first and third layers vary and that $T_0^1$=$T_0^3$ and $T_1^1$=$T_1^3$ to plot these graphs. Additionally, the angles $T_0^2$ and $T_1^2$ relative to the second layer were set equal to the optimal values found in the LW-LD2 case, which were $47.76^{\circ }$ and $36.46^{\circ }$, respectively. In contrast to the previous case, the complete gap map presents higher fundamental frequency values at the center of the design space.

**Table 10:** Optimal complete gap design angles varying the structural theory with the optimization constraint $\kappa _{{\text {lim}}}$ = 1.57 m^-1^

	ESL-TE1	ESL-TE3	LW-LD1	LW-LD2
$\langle T_0,T_1 \rangle ^1$ [^∘^]	$\langle -1.28, 0.54 \rangle$	$\langle -0.38,-1.09 \rangle$	$\langle -1.42, 0.26\rangle$	$\langle -1.76,-0.10\rangle$
$\langle T_0,T_1 \rangle ^2$ [^∘^]	$\langle 48.89, 19.74\rangle$	$\langle 53.76, 37.26\rangle$	$\langle 46.66, 37.14\rangle$	$\langle 47.76, 36.46\rangle$
$\langle T_0,T_1 \rangle ^3$ [^∘^]	$\langle -0.65, 2.32\rangle$	$\langle -2.28, 0.35 \rangle$	$\langle -1.21,-0.15\rangle$	$\langle -1.58,-0.10\rangle$
$f_1$ [Hz]	116.77	115.77	115.89	115.69

**Fig. 12:**
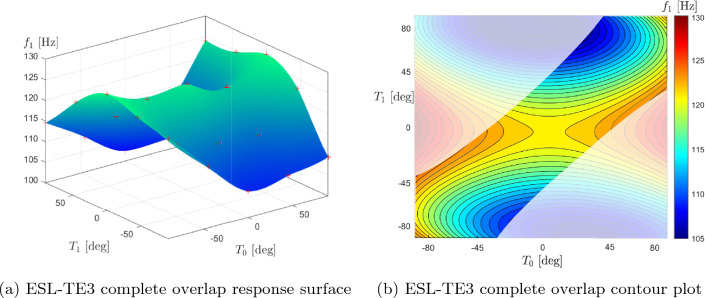
Response surface and contour plot of the fully clamped optimal complete overlap plate. The angles $T_0$ and $T_1$ of the second layer were kept fixed at $T_0^2=64.64^\circ$ and $T_1^2=21.97^\circ$; while the angles of the first and third layers were changed and assumed $T_0^1$=$T_0^3$ and $T_1^1$=$T_1^3$. The shaded area in the contour plot indicates the unfeasible design space

Table 11 shows the optimal results using the complete overlap strategy. As in the previous case, the range of the optimization variables $T_0$ and $T_1$ is also reduced to avoid the occurrence of singularities in the defect quantification process. As in the defect-free and complete gap cases, small variations in the optimal lamination angles and the maximum attainable fundamental frequency are observed as the structural theory changes. In particular, the first and third layers have very similar $T_0$ and $T_1$ angles. Therefore, plotting the response surface and the contour plot with the limitations due to the manufacturing process for the TE3 case is possible, as observed in Fig. 12. $T_0^1$ and $T_0^3$ have been set equal, as well as $T_1^1$ and $T_1^3$. The angles $T_0^2$ and $T_1^2$ have been fixed at 64.64^∘^ and 21.97^∘^, respectively. As previously stated, overlaps are modeled solely through ESL structural theories. In this manner, the thickness increments are compacted into an equivalent single layer, eliminating the need for additional expansions that would make the model computationally burdensome.

**Table 11:** Optimal complete overlap design angles varying the structural theory with the optimization constraint $\kappa _{{\text {lim}}}$ = 1.57 m^-1^

	ESL-TE1	ESL-TE3	ESL-TE4	ESL-TE6
$\langle T_0,T_1 \rangle ^1$ [^∘^]	$\langle 35.36,-6.16 \rangle$	$\langle 35.49,-5.93 \rangle$	$\langle 37.48,-5.33\rangle$	$\langle 35.36,-6.15\rangle$
$\langle T_0,T_1 \rangle ^2$ [^∘^]	$\langle 65.25,21.75\rangle$	$\langle 64.64, 21.97\rangle$	$\langle 63.18, 19.71\rangle$	$\langle 65.25, 21.75\rangle$
$\langle T_0,T_1 \rangle ^3$ [^∘^]	$\langle 33.37, -8.50\rangle$	$\langle 34.29,-9.42\rangle$	$\langle 31.57,-8.27\rangle$	$\langle 33.37,-8.49\rangle$
$f_1$ [Hz]	124.68	123.20	122.97	123.34

**Fig. 13:**
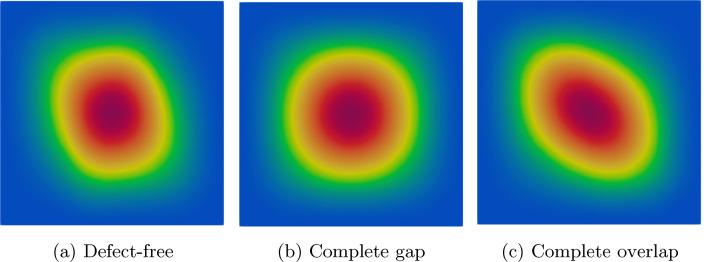
First mode shape in optimized configurations

Furthermore, it is observed in Tables 9, 10 and 11 that the ESL-TE 1 theory of structures, similar to the well-known FSDT, is the only one providing a slightly higher natural frequency. This occurs because—albeit relatively thin—shear effects are not negligible for the present composite plate. Also, no shear correction factors are used in this paper, even for ESL-TE 1 model although it should be advisable for first-order shear deformation theories, see [43]. Note that high-order models do not require corrections and they can deal naturally with high shear deformability demanded by the structures subject of the study. For further details on the effect of the structural theory on the natural frequencies of laminated structures, see [44].

Figure 13 illustrates the variation of the first modal form in the optimized defect-free, complete gap, and complete overlap cases. The shape of the first mode changes when defects are modeled and included in the optimization loop. Therefore, it is conceivable to consider the modal form for the problem of interest rather than focusing solely on the natural frequency.

## Conclusion

This paper presented the fundamental frequency optimization framework for tow-steered laminates, including gap and overlap defects and manufacturing process constraints. The flaws modeling was accomplished by coupling the DLM for defect mapping and quantification and the CUF-based FE analysis. It is observed that the optimal fiber paths differ depending on the modeling condition, i.e., defect-free or with fabrication flaws. In addition, the fact that a quasi-symmetric lay-up is retrieved is remarkable. That is, $\langle T_0^1,T_1^1\rangle \approx \langle T_0^3,T_1^3\rangle$. It is also important to note that the first and third layers of the optimal complete gap strategy have an almost unsteered design. The results showed that the complete gap strategy results in a lower maximum fundamental frequency, while the complete overlap strategy increases the maximum achievable fundamental frequency. Furthermore, as discussed in [32], the structural theory exhibits a weak dependency on the optimal $T_0$ and $T_1$ angles for the first natural frequency analysis, even when the manufacturing signature is included in the numerical model, as demonstrated by Tables 9, 10 and 11. As explained previously, only the ESL-TE1 models exhibit slightly higher natural frequencies.

**Fig. 14:**
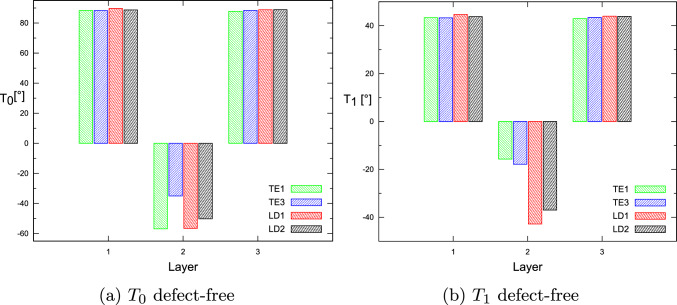
For the defect-free condition, the optimal $T_0$ and $T_1$ angles vary according to the structural theory for each layer

**Fig. 15:**
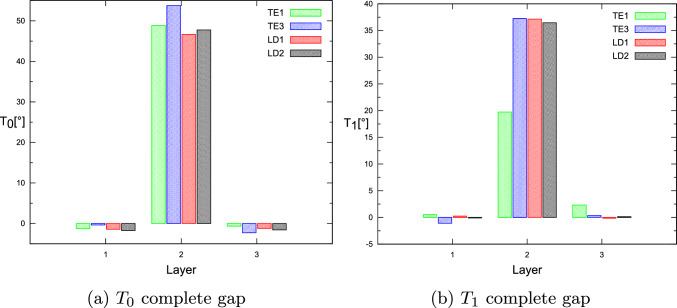
For the complete gap condition, the optimal $T_0$ and $T_1$ angles vary according to the structural theory for each layer

**Fig. 16:**
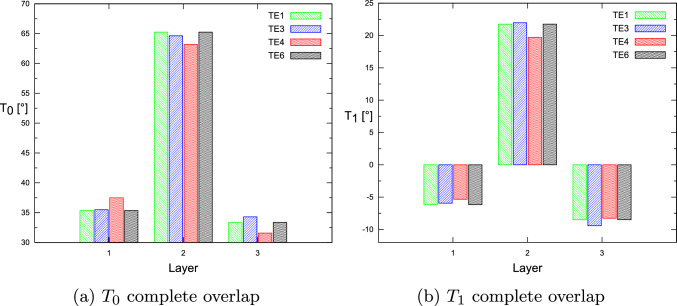
For the complete overlap condition, the optimal $T_0$ and $T_1$ angles vary according to the structural theory for each layer

Specifically, Fig. 14 summarizes the optimal angles $T_0$ and $T_1$ as the structural theory employed in the defect-free case varies. Only the angle $T_1^2$ is shown to be different from ESL to LW models. Figure 15 shows the angles of the optimum $T_0$ and $T_1$ in the case of a complete gap under different structural theories. Significant variations are only observed for the angles $T_1^2$ and $T_1^3$ relative to the ESL-TE1 model. Lastly, Fig. 16 displays the optimal angles $T_0$ and $T_1$ in case of complete overlap under different structural theories. No significant variations in the optimum angle exist when structural theory is modified.

The study demonstrates that the complete gap model results in an average 3% decrease in the first frequency compared to the defect-free condition. On the other hand, the complete overlap condition leads to a 2.70% increase in the maximum fundamental frequency achievable. The complete gap optimal design exhibits a smaller defect area than the complete overlap optimized solution as resin-rich areas reduce plate stiffness, whereas overlaps increase it.

As illustrated in Fig. 13, the shape of the first mode varies when defects are introduced. Thus, it would be interesting to investigate the modal shape optimization rather than focusing solely on the natural frequency in future investigations. Furthermore, the optimization framework presented in this work can be applied to investigate multi-objective optimization and mass-minimization problems in future research.

Furthermore, it has been shown that the use of VAT laminates can result in an increase in natural frequency of up to 10% compared to conventional straight fiber laminates. Considering only the limitations imposed by the maximum curvature radius achievable by the AFP machine, an increase in the fundamental frequency of approximately 3.4% is observed. When only gaps are considered, similar natural frequencies are observed between classic composites and those with curved fibers. Lastly, an increase of approximately 6.8% in the fundamental frequency is observed in the case of only overlaps. However, it is also necessary to compare this slight increase with the weight variations defects can cause. Future studies will focus on multi-objective optimization problems, considering mass variation. Similarly as [26], the results presented in this paper indicate a limited increase in the first natural frequency compared to classical laminates. This is due to the constraints of the manufacturing process and the simple geometry considered. Future investigations will focus on applying the optimization framework described in this manuscript to more complex models.

In conclusion, this study demonstrated that a dense FE mesh is not required to map the defect area accurately. Instead, high-order models based on CUF can be used to maintain larger elements and increase the order of through-the-thickness expansions without compromising the accuracy of the results.

## Data Availability

Data sets generated during the current study are available from the corresponding author on reasonable request.

## List of Symbols

$\mathbf {\widetilde{C}}$: Material stiffness matrix, [Pa]
$$\textbf{D}$$: Differential operator
$F_{\tau },F_s$: Through-the-thickness expansion functions
$f_1$: Fundamental frequency, [Hz]
$$\textbf{K}$$: Stiffness matrix
$$\textbf{M}$$: Mass matrix
$N_i,N_j$: Finite element shape functions
$\textbf{q}$: Unknown nodal vector
$T_0,T_1$: Fiber path angle parameters, [^∘^]
$$\textbf{u}$$: Displacement vector
$$\mathbf {\epsilon }$$: Strain tensor
$\theta$: Fiber angle orientation, [^∘^]
$$\kappa$$: Curvature, [m^-1^]
$$\rho$$: Density, [kg/m^3^]
$$\mathbf {\sigma }$$: Stress tensor
$\phi$: Fiber path rotation angle, [^∘^]

## Subscripts

ext: External
ine: Inertia
int: Internal
max: Maximum
min: Minimum
